# A unified voice for evidence-based psychiatry: a global response to the WHO guidance on ECT

**DOI:** 10.1007/s00406-026-02254-6

**Published:** 2026-04-28

**Authors:** Alexander Sartorius, David Zilles-Wegner

**Affiliations:** 1 Department of Psychiatry and Psychotherapy, Central Institute of Mental Health (CIMH), Medical Faculty Mannheim, University of Heidelberg, Square J 5, D-68159 Mannheim, Germany; 2 German Center for Mental Health (DZPG), Partner Site Mannheim/Heidelberg/Ulm, 68159 Mannheim, Germany; 3 Department of Psychiatry and Psychotherapy, University Medical Center Göttingen, 37075 Göttingen, Germany

The publication of the World Health Organization (WHO) *Guidance on Mental Health Policy and Strategic Action Plans* marked an important moment in global mental health policy. Its emphasis on rights-based, person-centered, and recovery-oriented care reflects principles that are widely shared across modern psychiatry. However, alongside these commendable aims, the guidance has raised substantial concern within the scientific community—particularly regarding its portrayal of biological treatments, and most notably electroconvulsive therapy (ECT) [1].

In response, an unprecedented joint statement by the World Psychiatric Association (WPA), the American Psychiatric Association (APA), the European Psychiatric Association (EPA), and the Global Expert Task Force on ECT (GET-ECT) has been published [2]. This coordinated international effort represents a remarkable and arguably historic moment: never before have these leading psychiatric organizations spoken with such a unified voice on a specific clinical and policy issue. The significance of this concerted action cannot be overstated.

At the core of the joint statement lies a fundamental concern: that the WHO guidance contains scientifically inaccurate and misleading claims about ECT, portraying it as inherently dangerous and advocating for its restriction or even prohibition. Such assertions stand in stark contrast to decades of empirical research and clinical experience. ECT is a well-established, evidence-based intervention, with demonstrated efficacy in severe and treatment-resistant psychiatric conditions, including major depression, psychotic disorders, and catatonia [3]. Moreover, contemporary data indicate that ECT is associated with reduced all-cause mortality and suicide risk, while serious adverse events are rare [4, 5].

Equally important is the growing body of neurobiological research suggesting that ECT induces neuroplastic changes rather than structural brain damage. Claims to the contrary, which are often based on subjective and anecdotal reports of permanent “memory loss,” are not evidence-based, but nonetheless serve to perpetuate prejudice and stigma surrounding ECT. As has been argued elsewhere [6], such misconceptions are not merely academic—they have tangible consequences. Misrepresentation of ECT may lead to reduced availability, delayed treatment, and ultimately preventable morbidity and mortality among severely ill patients.

The debate surrounding ECT also reflects a broader tension in contemporary psychiatry: the balance between biological, psychological, and social models of mental illness. The WHO guidance criticizes an “over-reliance on the biomedical approach” and calls for “holistic” treatments emphasizing social and structural determinants of mental health. This perspective risks replacing one form of reductionism with another as biological mechanisms and treatments (including all types of psychopharmacology) are implicitly marginalized. As has been noted in critical analyses [7], a comprehensive understanding of mental disorders must remain firmly grounded in a biopsychosocial framework. Important to note, this does not mean that all patients affected by severe psychiatric disorders benefit to the same extent from psychotherapy, social interventions, pharmacological treatment, or neurostimulation. Rather, we need to gain a better understanding of which patients benefit from which individualized treatments. Tailored treatment selection based on yet to validate clinical and biological predictors or moderators of response will hopefully be the near future of psychiatric care. ECT exemplifies this complexity. Its mechanisms of action likely involve a multitude of neurobiological processes, including seizure termination pathways and subsequent reparative and neuroplastic responses. At the same time, its clinical application is embedded in ethical, legal, and psychosocial contexts. Any attempt to reduce ECT to a single dimension—whether biological or sociopolitical—fails to capture its full clinical reality.

Against this backdrop, the joint statement by WPA, APA, EPA, and GET-ECT represents more than a defense of a specific treatment modality. It is, fundamentally, a call for scientific integrity in global mental health policy. The authors explicitly endorse the WHO’s overarching goals but emphasize that these must be aligned with robust empirical evidence and clinical expertise. Importantly, they also highlight the perspectives of patients and caregivers, whose lived experiences often contradict the negative portrayals found in the WHO guidance.

This unified position also underscores the importance of international collaboration in psychiatry. Mental health care is increasingly shaped by global narratives and policy frameworks. When these frameworks diverge from the evidence base, coordinated responses are essential. The present joint statement demonstrates that such coordination is possible—and effective.

At the same time, the discussion initiated by the WHO guidance offers an opportunity for reflection at the national level. In Germany, as in many other countries, psychiatric care is delivered within a pluralistic landscape that includes biological psychiatry, psychotherapy, and social psychiatry. Each of these approaches to treating mental illness has its own merits and potential benefits for patients. However, communication and collaboration between these domains is often less integrated than it should be. If we truly want what’s best for our patients, the various providers and experts need to work together more effectively (and trustingly) to create added value for mental health care and the individual patient.

Importantly, this is not merely a matter of professional alignment. A stronger, interdisciplinary consensus would allow for a more effective articulation of the biopsychosocial model in international discourse. Rather than opposing biological and psychosocial approaches, psychiatry should present them as complementary components of a comprehensive treatment framework. In doing so, it can more convincingly advocate for policies that reflect the full complexity of mental health care.

The current situation also highlights the need for proactive engagement with global health organizations. Waiting to respond to finalized policy documents may be insufficient. Instead, early dialogue, participation in guideline development, and the inclusion of a broad range of stakeholders—including clinicians, researchers, and patients—are crucial. The unprecedented collaboration demonstrated in the joint statement may serve as a model for such future efforts.

In conclusion, the joint response of WPA, APA, EPA, and GET-ECT to the WHO guidance represents a landmark moment in international psychiatry. It reaffirms the importance of evidence-based practice, combats the perpetuation of stigma and misinformation, and demonstrates the power of coordinated professional advocacy. At the same time, it serves as a reminder that the future of mental health care depends not only on scientific advances but also on our ability to communicate, collaborate, and engage—both within our own disciplines and on the global stage.

Only through such concerted efforts can we ensure that mental health policy remains aligned with the best available evidence and, ultimately, with the needs of the patients we serve.
